# Differential Proteomics of *Helicobacter pylori* Isolates from Gastritis, Ulcer, and Cancer Patients: First Study from Northwest Pakistan

**DOI:** 10.3390/medicina58091168

**Published:** 2022-08-28

**Authors:** Syed Ali Raza Shah, Hazir Rahman, Muhammad Qasim, Muhammad Safwan Akram, Yasemin Saygideger, Nanda Puspita, Burcu Saygıdeğer Demir, Khalid J. Alzahrani, Muhammad Fayyaz ur Rehman, Fuad M. Alzahrani, Mohamed A. Alblihd

**Affiliations:** 1 Department of Microbiology, Abdul Wali Khan University, Mardan 23200, KP, Pakistan; 2 Department of Microbiology, Kohat University of Science and Technology, Kohat 26000, KP, Pakistan; 3 National Horizons Centre, Teesside University, Darlington DL11HG, UK; 4 School of Health & Life Sciences, Teesside University, Middlesbrough TS1 3BX, UK; 5 Department of Pulmonary, Cukurova University School of Medicine, 01330 Adana, Turkey; 6 Department of Translational Medicine, Institute of Health Sciences, Cukurova University, 01330 Adana, Turkey; 7 Department of Biotechnology, Institute of Natural and Applied Sciences, Cukurova University, 01330 Adana, Turkey; 8 Department of Clinical Laboratories Sciences, College of Applied Medical Sciences, Taif University, Taif 21944, Saudi Arabia; 9 Institute of Chemistry, University of Sargodha, Sargodha 40100, Pakistan; 10 Department of Medical Microbiology, College of Medicine, Taif University, P.O. Box 11099, Taif 21944, Saudi Arabia

**Keywords:** *H. pylori*, proteomics, gastric patients, mass spectrometry

## Abstract

*Background and Objective*: *Helicobacter pylori* is a human-stomach-dwelling organism that causes many gastric illnesses, including gastritis, ulcer, and gastric cancer. The purpose of the study was to perform differential proteomic analysis on *H. pylori* isolates from gastritis, ulcer, and gastric cancer patients. *Materials and Methods*: *H. pylori* was isolated from antrum and fundus biopsies obtained from patients who visited the Department of Gastroenterology. Using nano-LC-QTOF MS/MS analysis, differentially regulated proteins were identified through proteome profiling of pooled samples of *H. pylori* isolated from gastritis, ulcer, and gastric cancer patients. Antigenic scores and cellular localization of proteins were determined using additional prediction tools. *Results*: A total of 14 significantly regulated proteins were identified in *H. pylori* isolated from patients with either gastritis, ulcer, or gastric cancer. Comparative analysis of groups revealed that in the case of cancer vs. gastritis, six proteins were overexpressed, out of which two proteins, including hydrogenase maturation factor (hypA) and nucleoside diphosphate kinase (ndk) involved in bacterial colonization, were only upregulated in isolates from cancer patients. Similarly, in cancer vs. ulcer, a total of nine proteins were expressed. Sec-independent protein translocase protein (tatB), involved in protein translocation, and pseudaminic acid synthase I (pseI), involved in the synthesis of functional flagella, were upregulated in cancer, while hypA and ndk were downregulated. In ulcer vs. gastritis, eight proteins were expressed. In this group, tatB was overexpressed. A reduction in thioredoxin peroxidase (bacterioferritin co-migratory protein (bcp)) was observed in ulcer vs. gastritis and cancer vs. ulcer. *Conclusion*: Our study suggested three discrete protein signatures, hypA, tatB, and bcp, with differential expression in gastritis, ulcer, and cancer. Protein expression profiles of *H. pylori* isolated from patients with these gastric diseases will help to understand the virulence and pathogenesis of *H. pylori*.

## 1. Introduction

*Helicobacter pylori* is a microaerophilic, gram-negative bacterial pathogen infecting more than half of the global population [1]. The incidence of *H. pylori* ranges from 30 to 50% in developed countries, while the incidence is much higher in developing countries, ranging from 85 to 95% [2,3,4]. It induces an inflammatory response in the stomach by colonizing the gastric epithelium [5,6]. The pathogen persists for decades within the host and increased infection is associated with several diseases ranging from mild to severe infections such as autoimmune atrophic gastritis, duodenal ulcer, and gastric cancer [7,8,9,10]. Gastric ulcer develops in 10–20% of infected individuals and 1–3% may develop gastric cancer [11]. The risk of gastric cancer is 3–6-fold higher in *H. pylori*-infected individuals compared to non-infectious individuals [12,13].

The prevalence of *H. pylori* in Pakistan is very high (81%) when compared to other South Asian countries [14]. This high prevalence is primarily due to factors such as dietary habits and co-infection with other gastrointestinal pathogens [15]. Numerous proteomic and genomic studies have identified pathogenic variants responsible for causing a clinical outcome in the host; however, genetic diversity leading to geographical variations of the pathogen is considered responsible for different phenotypes and disease onset [16,17]. It is well reported that genetic diversity augments protein abundance, mainly via transcriptional and post-transcriptional signaling [18,19]. Proteomic investigation of the pathogen is a promising tool to obtain protein profiles associated with a distinct clinical outcome. Further proteome profiling of *H. pylori* is important to correlate epigenetic alterations with gene regulation and virulence mechanism. Similarly, protein signature among gastric-disease-causing *H. pylori* isolates validate the correlation between pathogen and disease pattern [20,21,22].

Investigation of transition from a mild to a severe form of the disease at the expression level is still elusive, and the molecular pathway is not yet understood. This work investigated protein expression profiles of *H. pylori* isolated from gastritis, gastric ulcer, and gastric cancer patients using LC-MS/MS analysis. Findings of the study may help to understand pathogenesis of *H. pylori*.

## 2. Materials and Methods

This study was conducted at the Department of Microbiology, Abdul Wali Khan University Mardan between 2019 and 2021. The study was approved by the ethics committee of Hayatabad Medical Complex Peshawar (reference number:370/HEC/B&PSC/2020) for use of human subjects, the approval date is 28 November 2020. Data were collected through questionnaire and informed written consent was taken from the patients (*n* = 150) visiting the endoscopy section of the Gastrointestinal Department.

### 2.1. Identification of Helicobacter pylori

**Figure 1:**
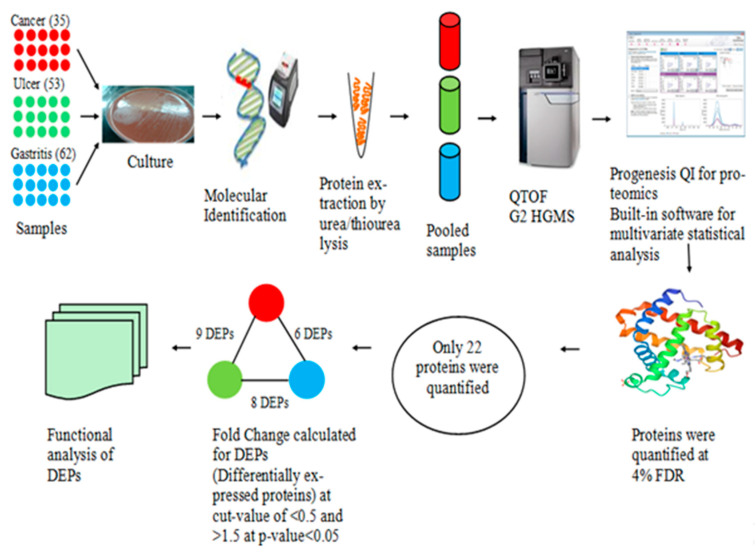
Schematic diagram of protein profiling.

The schematic representation of the methodology is given in Figure 1. For confirmation of *Helicobacter pylori* infection, fresh biopsy samples from patients with gastric cancer (*n* = 35), gastric ulcer (*n* = 53), and gastritis (*n* = 62) were collected. About 4 mm of tissue samples from the antrum of the stomach were taken in a sterilized Petri plate and cut into pieces using a sterile scalpel blade. Biopsy samples were cultured on Columbia blood agar base (Oxoid, Hampshire, UK), supplemented with 5% sheep blood, using DENT (Oxoid, Hampshire, UK) and CampyGen sachet (Thermo Fischer Scientific, Warrington, UK) and was morphologically and phenotypically identified. Biochemical identification was carried out using oxidase, catalase, and urease tests [23]. The findings were confirmed through PCR targeting species-specific 16S rRNA genes using primers (F-5′-GCGACCTGCTGGAACATTAC-3′), R (5′-CGTTAGCTGCATTACTGGAGA-3′).

### 2.2. Sample Preparation and Peptide Sequencing

PCR-positive *H. pylori* was sub-cultured in thioglycolate broth (Oxoid, Hampshire, UK) with CampyGen sachet for 72 h in an anaerobic jar. The broth was centrifuged at 4000× *g* for 20 min at 4 °C and a pellet was obtained. For label-free proteomics, proteins were extracted by resuspending the pellet in 800 μL of lysis buffer (Urea 7M, Thiourea 2M, 4% CHAPS and 1% DTT) and then 500 µL of protein solution was desalinized using Amicon Ultra-0.5 mL 3K-NMWL filter devices (Merck Millipore Corporation, Darmstadt, Germany). The Bradford method was used to determine the final protein concentration. A measure of 500 μg of proteins were denatured with 0.1% RapiGest (RapiGest SF, Waters, Milford, CT, USA), then reduced with dithiothreitol (final concentration 5 mM) and alkylated with iodoacetamide (final concentration of 15 mM). For in-solution digestion, 60 µg of proteins from 10 pooled samples of each group (gastritis, gastric ulcer, and gastric cancer) was used. Trypsin digestion was performed with sequencing Grade Modified Trypsin (Thermo Scientific™, Waltham, MA, USA) at a 1:100 (*w*/*w*) enzyme: protein ratio. After digestion, 1% (*v*/*v*) of trifluoroacetic acid was added to hydrolyze the RapiGest. The digested peptides were filtered using 0.2 µm spin filter (Corning, New York, NY, USA). Peptide sequencing was performed on QTOF G2 HDMS (Waters, Milford, CT, USA) at National Horizons Centre, Teesside UK. Chromatogram of the peptide fragments were acquired by reverse-phase ultraperformance liquid chromatography (Acquity UPLC H-Class System, Waters, Milford, CT, USA). The separation was performed through an ACQUITY UPLC HSS T3 2.1 mm × 100 mm column, using a binary gradient from 2 to 85% of acetonitrile with 1% formic acid (*v*/*v*) for 62, at a flow rate of 250 μL/min. Data-independent scanning (MSE) experiments were performed by switching between low and elevated collision energies (25–50 eV), and a scan time of 0.5 s was used for low- and high-energy scans from *m*/*z* 50 to 2000.

### 2.3. Data Analysis

The raw files from QTOF mass spectrometer were analyzed by Progenesis QI for proteomics (Waters, Milford, CT, USA). The reference protein sequences were downloaded from NCBI database to search MS/MS spectra against the reference sequence. A false discovery rate of 4% was selected. Peptide tolerance and fragment tolerance were auto-selected. Proteins with 1 or more peptides, 3 fragments per peptide or 7 fragments per protein identified were considered quantifiable. Protein fold change was calculated using *t*-test, and cut-values of <0.5 and >1.5 at *p*-value < 0.05 were considered significantly differentiated proteins. FASTA sequences from Uniprot (https://www.uniprot.org, accessed on 22 April 2022) for significantly differentiated proteins were downloaded and was uploaded on STRING version 11.5 (https://string-db.org, accessed on 16 June 2022, Version number 11.5, Mardan, Pakistan) for protein–protein interaction networks and functional enrichment analysis. CELLO2GO web server (http://cello.life.nctu.edu.tw/cello2go/, accessed on 16 June 2022) was used for the subcellular localization of differentiated proteins (Yu et al., 2014). The VaxiJen: Prediction of Protective Antigens and Subunit Vaccines (version 2.0) webserver was used for potent antigenicity prediction of differentially expressed proteins. InteractiVenn, an online tool [24], was used for plotting Venn diagrams of overlapping proteins in three comparisons. Finally, protein fold change of overlapping proteins was plotted to compare variation in the expression of proteins from one disease to another and their probable interaction with each other.

## 3. Results

**Figure 2:**
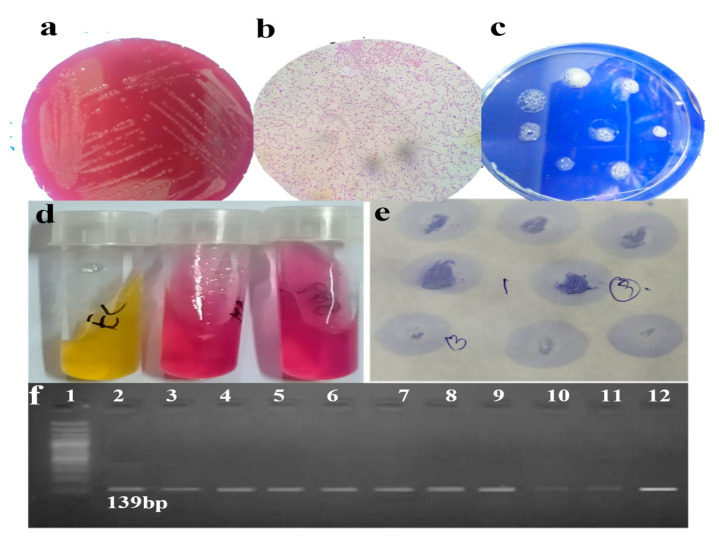
Identification of *H. pylori*. (**a**) Colonies on Columbia agar; 1–2 mm in diameter, small, translucent, and non-hemolytic colonies on agar plate were identified as *H*. *pylori*. (**b**) Gram staining: microscopic image of negatively stained *H. pylori*. (**c**) Catalase assay: appearance of bubbles indicates positive catalase reaction. (**d**) Urease test: a pink tint in the medium indicates a positive reaction, while yellow indicates negative control. (**e**) Oxidase test: positive test is indicated by a rich purple color produced within 5–10 s. (**f**) Species-specific 16S rRNA gene amplification: 100 bp marker is used (shown in well 1) and samples amplified at 139 bp are shown in well 2–12).

The isolation of *H. pylori* from the biopsy samples of gastric patients was performed through culturing. Results of cultured samples and subsequent microscopical examination along with biochemical and PCR amplification are depicted in Figure 2.

Among the included subjects (*n* = 150), 42.7% of patients were more than 50 years of age, and 54% of patients were females and 46% of patients were males. Of the subjects, 76.7% had no ulcer complications and a large number of subjects, i.e., 41.3%, were suffering from gastritis, followed by 35.3% of ulcer patients. Demographic factors are summarized in Table 1.

Proteome investigation of *H. pylori* identified a total of 22 proteins (Supplementary Table S1) that comprised 6 significantly different proteins in cancer compared to gastritis with a fold change ranging from −2.52 to +4.81, whereas 9 proteins were significantly regulated in cancer compared to ulcer, with a fold change ranging from −2.66 to +10.19, while 8 proteins were significantly regulated in ulcer compared to gastritis, ranging from −6.16 to +4.18. The most significantly regulated protein was thioredoxin peroxidase, which was found to be downregulated with a value of −6.16 from gastritis to ulcer, and −10.19 from ulcer to cancer patients. Details of identified proteins are tabulated in Table 2.

Venn diagram shows no overlapping protein among three groups; however, three different proteins were found to be overlapping between the two groups. Compared to gastritis, both in ulcer and cancer, hypA protein showed an upregulation which is involved in Nickle cation binding and plays a role in Pathogenesis.

**Figure 3:**
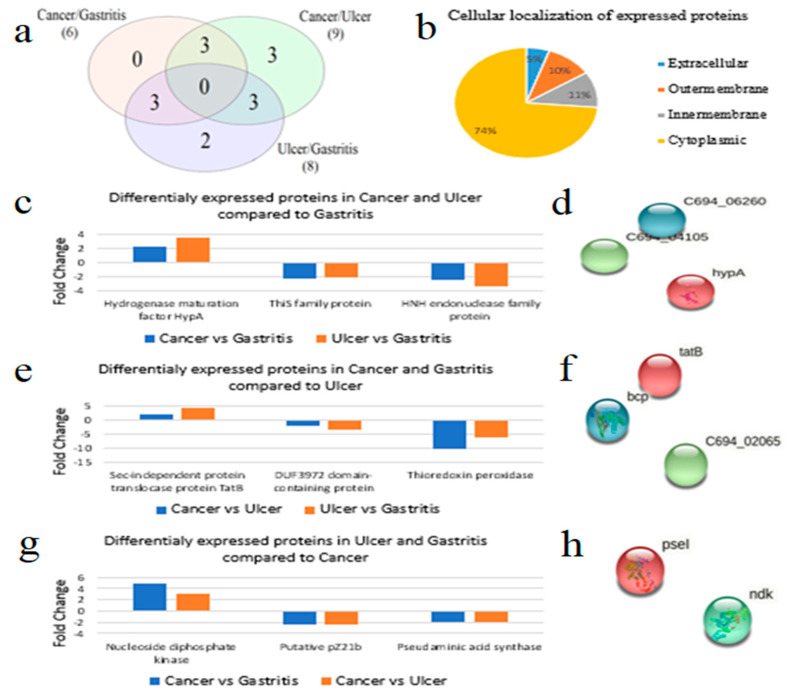
Profile of differentially regulated proteins of *H. pylori*. (**a**) Venn diagram of overlapping proteins; common proteins from each comparison is shown by union of all three comparisons; (**b**) cellular localization of expressed proteins; (**c**–**h**) comparison of common differentially expressed proteins in two groups and their interaction by STRING; left side depicts bar plots of common proteins comparing their fold change values in two groups and right side depicts their interaction with one another.

This family protein, which is an unreviewed protein in the Uniprot database, was downregulated with disease severity, while HNH endonuclease family protein showed higher levels of suppression in ulcer compared to cancer. Similarly, comparison of gastritis and cancer with ulcer indicated a suppression of thioredoxin peroxidase in the case of cancer, and overexpression in the case of gastritis. A similar trend was observed in the case of DUF3972-domain-containing proteins, but there is a small degree of decrease in protein downregulation compared to thioredoxin peroxidase. Sec-independent protein translocase protein tatB showed a reverse trend compared to those of the other two proteins in the group. In the third comparison of overlapping proteins of cancer vs. ulcer and cancer vs. gastritis, nucleoside diphosphate kinase expression was lower in both gastritis and ulcer, indicating an increase in kinase activity in severe disease forms. An opposite trend was observed in the case of putative PZ21b protein and pseudaminic acid synthase protein, both of which are antigenic proteins. A Venn diagram of union and cellular localization of expressed proteins along with differential expression of overlapping proteins in each group is illustrated in Figure 3.

**Figure 4:**
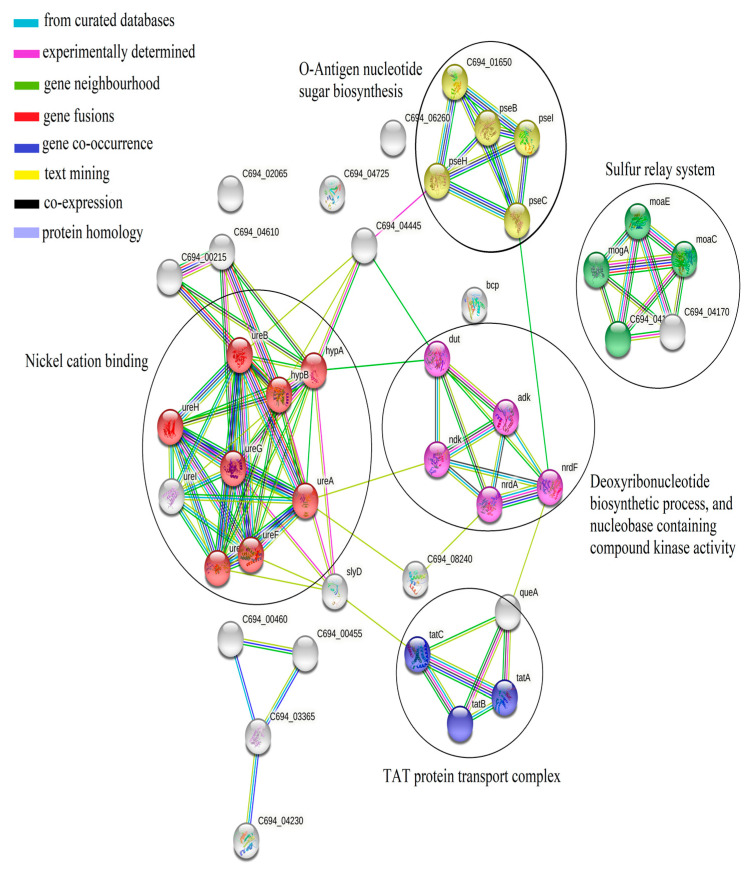
Interactomics map of differentially expressed proteins of *H. pylori*. Red nodes show proteins involved in nickel cation binding. Blue nodes show proteins that have role in TAT protein transport complex. Proteins of O-antigen nucleotide sugar biosynthesis are indicated by yellow nodes, while proteins of the sulfur relay system are shown by green color nodes. Purple node exhibits proteins involved in biosynthesis of deoxyribonucleotide and kinase activity. Interaction types are shown by different colored edges, as indicated by legends.

Protein–protein interaction by STRING database showed higher percentage of identities matched with *H. pylori* 26695 strain, but no interaction was found among these proteins (Figure 3). However, some of these proteins are involved in important molecular processes and pathways. For instance, hypA is involved in nickel cation binding, tatB is a cellular component of the TAT protein transport complex, pseI is a part of O-Antigen nucleotide sugar biosynthesis, and ThiS family protein (C694_04105), which is a Molybdopterin-converting factor, as a small subunit, is involved in the sulfur relay system (Figure 4). Cellular localization of those proteins represented 14 (74%) as cytoplasmic, 2 (11%) inner membrane, 2 (10%) outer membrane, and 1 (5%) extracellular protein. Further exploring antigenicity of the proteins at threshold level of 0.4, 7 out of 14 proteins were predicted as antigenic (Table 3).

**Figure 5:**
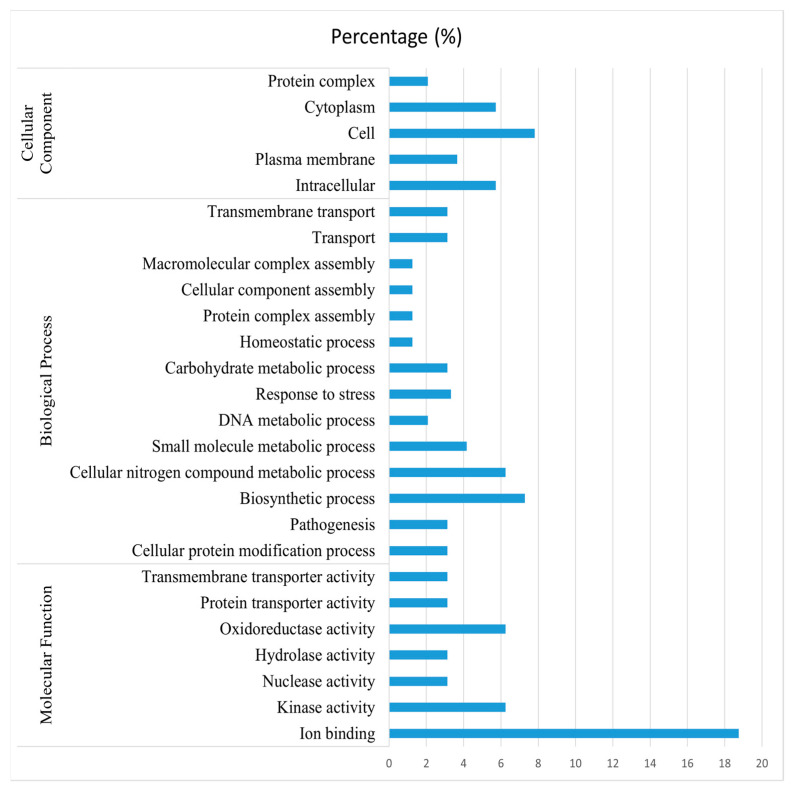
Cellular, biological, and molecular functions of differentially expressed proteins of *H. pylori*.

Investigating the role of expressed proteins revealed that they serve as cellular components such as protein complexes, cytoplasm, cell membranes, and intracellular components. Furthermore, these proteins are involved in a variety of biological processes (transmembrane transport, macromolecular complex assembly, response to stress, pathogenesis, etc.), as well as molecular functions such as oxidoreductase activity, hydrolase activity, nuclease activity, etc. (Figure 5).

## 4. Discussion

In the current study, the proteome profile of *H. pylori*, infecting the Pakistani population and causing a sequel of diseases from the milder form, i.e., gastritis, to the severe form, i.e., ulcers, and ultimately the fatal form, i.e., gastric cancer, was investigated. There are several epidemiological and genetic studies on *H. pylori* reported from Pakistan; however, there is a severe lack of molecular data, such as those addressed in this proteomic investigation. We identified proteins responsible for different disease phenotypes through differential protein expression utilizing nano LC-QTOF analysis.

For proteome profiling, 10 pooled samples of *H. pylori* each from gastritis, ulcer, and cancer were processed. Among the identified proteins, hypA, which is responsible for nickel cation binding, was upregulated in cancer and ulcer. Nickel is a virulence determinant because it is a co-factor of urease, which is required for homing in the stomach by resisting acidity. A previous study indicated a 50-times-higher concentration of nickel in *H. pylori* compared to *E. coli* [22]. Therefore, a supply of nickel is crucial for its survival in the stomach. In our study, the overexpression of hypA protein in cancer and ulcer might increase nickel binding and urease activity, and this has a role in disease progression.

Pseudaminic acid was upregulated in cancer vs. ulcer, but downregulated in cancer vs. gastritis. It is reported that flagellar glycosylation is vital for flagellar function [23,24], and the inactivation of genes responsible for post translational modifications, such as glycosylation, may result in the inactivation of flagella [25,26]. O-linked glycosylation caused by synthesis of pseudaminic acid (Pse) is crucial for functional flagella. In addition, Pse biosynthesis aids in the production of virulence factors such as urease and lipopolysaccharide (LPS) [27,28]. It suppresses host immune response by masking the epitope of antigens of outer membrane proteins [29]. Previous genetic studies have reported elimination of functional flagella due to the inactivation of the pseI gene of *H. pylori*, ultimately resulting in reduced motility [30,31]. Increased expression of PseI in cancer vs. ulcer and decreased expression in cancer vs. gastritis might reflect its role in the initial and advanced stages of disease onset caused by *H. pylori*.

Another enzyme expressed with the highest value of suppression is thioredoxin peroxidase. Protein–protein interaction analysis of thioredoxin peroxidase showed bacterioferritin co-migratory protein (bcp) in *H. pylori* 26695 strain as the most identical protein with an identity score of 98.7%. Bacterioferritin co-migratory protein (bcp) belongs to the peroxiredoxin family and is commonly known as thiol peroxidase [32]. The peroxidase enzyme utilizes reduced thioredoxin as an electron donor for its substrate catalytic reduction. Thioredoxins and thiol peroxidases play a role in resistance to bactericidal reactive oxygen species and reactive nitrogen species [33]. However, bcp is shown to be involved in the removal of fatty acid hydroperoxides generated by oxidative stress or by metabolism [34]. Further mechanistic studies to explore its role in infection would help to understand its inverse relationship with the severity of the disease.

One of the protective effector molecules is nucleoside diphosphate kinase (ndk) secreted by intracellular microorganisms during tissue colonization in the host. This effector molecule modulates signaling of host-derived small danger molecules [35,36], establishing persistent infections in the host [37]. Ndk-Purinergic signaling, by which ndk reduces extracellular purine signaling molecules such as extracellular ATP, is the primary target by which it modulates host immune response. Secretion of these effector molecules by *H. pylori* interact with oncogenic pathways and can induce carcinogenesis [38]. It is of great importance due to multiple immune invasion strategies during infection. In our study, upregulation of ndk in cancer-causing *H. pylori* might have a potential role in gastric adenocarcinoma.

The twin-arginine translocation (Tat) pathway plays a remarkable role in the transport of folded proteins [39]. This pathway transports folded proteins in a secretory (sec)-independent manner [40] and is specifically involved in the export of virulence proteins. Location and activity of hydrogenase and catalase is affected with impaired Tat machinery in *H. pylori*. In the current study, the upregulation of tatB in cancer- and gastritis-causing *H. pylori* might have a potential role in the virulence mechanism of the pathogen.

The proteins expressed in our study were either directly playing a role in virulence, such as hypA, ndk, pseI, and bcp, by facilitating the pathogen in colonization within the acidic medium of the stomach, while tatB is involved in the translocation of virulence proteins. However, the specific role of tatB and bcp in pathogenesis is still unknown. Further studies about the specific role of these proteins in gastric pathologies would help to understand the molecular pathogenesis of *H. pylori*.

## 5. Conclusions

Differential proteome of the isolates revealed upregulation of hypA protein involved in bacterial colonization and tatB protein involved in protein translocation, while revealing downregulation of bacterioferritin co-migratory protein (bcp), that plays a role in resistance against reactive oxygen and nitrogen species. Functional characterization of the identified proteins would help to explain the pathogenesis and virulence mechanism of *H. pylori*.

## Figures and Tables

**Table 1:** Demographic data of biopsy patients.

Factors		*n* = 150	HP Positive *n* (%)	HP Negative *n* (%)	*p*-Value
Age group	1–25 years	11 (7.3)	5 (6.7)	6 (8.0)	0.949
	26–50 years	75 (50.0)	38 (50.7)	37 (49.3)	
	>50 years	64 (42.7)	32 (42.7)	32 (42.7)	
Gender	Female	81 (54.0)	35 (46.7)	46 (61.3)	0.072
	Male	69 (46.0)	40 (53.3)	29 (38.7)	
Ulcer complications	No	115 (76.7)	56 (74.7)	59 (78.7)	0.562
	Yes	35 (23.3)	19 (25.3)	16 (21.3)	
Endoscopic finding	Cancer	35 (23.3)	24 (32.0)	11 (14.7)	0.037
	Gastritis	62 (41.3)	29 (38.7)	33 (44.0)	
	Ulcer	53 (35.3)	22 (29.3)	31 (41.3)	
Biopsy sites	Antrum	91 (60.7)	55 (73.3)	36 (48.0)	0.000
	Corpus	43 (28.7)	19 (25.3)	24 (32.0)	
	Fundus	16 (10.7)	1 (1.3)	15 (20.0)	

**Table 2:** Up- and downregulated proteins analyzed with Q TOF mass spectrometry.

S. No.	Condition	Status	Accession No.	Protein Name	Mol. W * (Da)	Gene Name	MFC **
1	Cancer vs. Gastritis	Upregulated	GI:407224289	Hydrogenase maturation factor HypA	13,202	hypA	2.32
			GI:407225892	Nucleoside diphosphate kinase	15,318	ndk	4.81
		Downregulated	GI:407224220	ThiS family protein	8097	OUQ_1033	−2.23
			GI:407223472	HNH endonuclease family protein	11,209	OUQ_1426	−2.52
			GI:407223928	Putative pZ21b	16,656	OUQ_1226	−2.41
			GI:407225875	Pseudaminic acid synthase	38,076	pseI	−2.02
2	Cancer vs. Ulcer	Upregulated	Not available	Uncharacterized protein	16,179	OUQ_1153	2.22
			GI:407224758	Sec-independent protein translocase protein TatB	17,799	tatB	2.16
			GI:407223928	Putative pZ21b	16,656	OUQ_1226	2.33
			Not available	DUF3944 domain-containing protein	12,002	OUQ_0172	2.66
			GI:407225875	Pseudaminic acid synthase	38,076	pseI	2.03
		Downregulated	GI:407224184	Uncharacterized protein	11,580	OUQ_0997	−2.11
			GI:1661363461	DUF3972 domain-containing protein	22,550	OUQ_0609	−2.29
			GI:407225892	Nucleoside diphosphate kinase	15,318	ndk	−3.06
			Not available	Thioredoxin peroxidase	17,130	OUQ_0313	−10.19
3	Ulcer vs. Gastritis	Upregulated	GI:407224289	Hydrogenase maturation factor HypA	13,202	hypA	3.59
			GI:407224758	Sec-independent protein translocase protein TatB	17,799	tatB	4.18
			GI:407225788	Glycosyl transferase 11 family protein	17,194	OUQ_0267	3.48
		Downregulated	GI:1661363461	DUF3972 domain-containing protein	22,550	OUQ_0609	−3.49
			GI:407224220	ThiS family protein	8097	OUQ_1033	−2.06
			GI:407225957	Flagellar FliJ protein	16,751	OUQ_0438	−2.88
			Not available	Thioredoxin peroxidase	17,130	OUQ_0313	−6.16
			GI:407223472	HNH endonuclease family protein	11,209	OUQ_1426	−3.34

* Mol. W = molecular weight of proteins. ** MFC = maximum fold change.

**Table 3:** Antigenicity and VaxiJen Score of expressed proteins among *H. pylori* isolates.

Protein Name	VaxiJen Score	Antigenicity
Hydrogenase maturation factor HypA	0.3364	Non-antigenic
Nucleoside diphosphate kinase	0.3847	Non-antigenic
ThiS family protein	0.3488	Non-antigenic
HNH endonuclease family protein	0.9778	Antigenic
Putative pZ21b	0.5140	Antigenic
Pseudaminic acid synthase	0.4120	Antigenic
Uncharacterized protein	0.1738	Non-antigenic
Sec-independent protein translocase protein TatB	0.5732	Antigenic
DUF3944 domain-containing protein	0.4661	Antigenic
Uncharacterized protein	0.1528	Non-antigenic
DUF3972 domain-containing protein	0.3545	Non-antigenic
Thioredoxin peroxidase	0.4745	Antigenic
Glycosyl transferase 11 family protein	0.4499	Antigenic
Flagellar FliJ protein	0.3321	Non-antigenic

## Data Availability

Data will be made available on request.

## Supplementary Materials

The following supporting information can be downloaded at: https://www.mdpi.com/article/10.3390/medicina58091168/s1, Table S1: Proteins identified through Q-TOF mass spectrometry in *H. pylori* isolates.
