# Supplementary material for: Differential Proteomics of Helicobacter pylori Isolates from Gastritis, Ulcer, and Cancer Patients: First Study from Northwest Pakistan
Source: Medicina (Kaunas). 2022 Aug 28;58(9):1168. doi: 10.3390/medicina58091168 (PMC9500814; doi:10.3390/medicina58091168)
Supplement: Supplementary file 1 [file medicina-58-01168-s001.zip › medicina-1799329-supplementary.pdf]

Supplementary Table S1: Proteins identified through Q-TOF mass spectrometry in *H. pylori* isolates

S. No	Protein Name	Gene
1	Uncharacterized protein	OUQ_0997
2	Nucleoside diphosphate kinase	ndk
3	Thioredoxin peroxidase	bcp
4	Ribosome maturation factor RimM	rimM
5	DUF3972 domain-containing protein	OUQ_0609
6	Putative TRANSCRIPTIONAL REGULATOR	OUQ_0342
7	HNH endonuclease family protein	OUQ_1426
8	ThiS family protein	OUQ_1033
9	Flagellar FliJ protein	OUQ_0438
10	Putative pZ21b	OUQ_1226
11	Hydrogenase maturation factor HypA	hypA
12	Uncharacterized protein	OUQ_1153
13	Type I restriction-modification system_ M subunit	hsdM
14	Sec-independent protein translocase protein TatB	tatB
15	Uncharacterized protein	OUQ_1102
16	DUF3944 domain-containing protein	OUQ_0172
17	Putative 3'-5' exonuclease	OUQ_0544
18	Glycosyl transferase 11 family protein	OUQ_0267
19	D_D-heptose 1_7-bisphosphate phosphatase	gmhB
20	ddrB-ParB domain-containing protein (Fragment)	OUQ_1280
21	Pseudaminic acid synthase	pseI
22	AAA ATPase domain protein	OUQ_0379
